# Effect of Fecal Microbiota Transplantation on Arterial Stiffness in Alcohol-Related Liver Cirrhosis: A Prospective Pilot Study

**DOI:** 10.3390/life16040668

**Published:** 2026-04-14

**Authors:** Cristian Ichim, Adrian Boicean, Romeo Mihaila, Samuel Bogdan Todor, Paula Anderco, Victoria Birlutiu

**Affiliations:** Faculty of Medicine, Lucian Blaga University of Sibiu, 550169 Sibiu, Romania; cristian.ichim@ulbsibiu.ro (C.I.); romeo.mihaila@ulbsibiu.ro (R.M.); paula.anderco@ulbsibiu.ro (P.A.); victoria.birlutiu@ulbsibiu.ro (V.B.)

**Keywords:** alcohol-related liver disease, fecal microbiota transplantation, arterial stiffness, gut microbiota

## Abstract

Background: Alcohol-related liver disease is frequently associated with systemic vascular dysfunction and increased arterial stiffness. This may contribute to adverse clinical outcomes. Modulation of the gut microbiota through fecal microbiota transplantation (FMT) has emerged as a potential therapeutic strategy in liver cirrhosis, but its influence on vascular stiffness in humans remains insufficiently characterized. Methods: This prospective study evaluated arterial stiffness in patients with alcohol-related liver cirrhosis undergoing FMT. A control group received standard care. Vascular stiffness was assessed non-invasively using an oscillometric arteriograph based on pulse wave analysis. Measurements were performed at baseline and at one and three months after FMT under standardized conditions. The main indices assessed included aortic pulse wave velocity, augmentation index, ejection duration and return time. Direct microbiome sequencing and metabolomic profiling were not performed. Results: At baseline, the study and control groups had comparable vascular stiffness profiles. Only minor differences in selected hemodynamic parameters were observed. At one month after intervention, no statistically significant differences in arterial stiffness indices were observed between groups. Longitudinal analysis within the FMT group also showed no significant changes in direct markers of arterial stiffness across the three-month follow-up period. A non-significant tendency toward reduced ejection duration was noted. Conclusions: In patients with advanced alcohol-related liver cirrhosis, FMT did not produce measurable short-term improvements in arterial stiffness. These findings suggest that short-term vascular effects of microbiota modulation may be difficult to detect in patients with advanced alcohol-related liver cirrhosis. Larger studies including earlier-stage patients, longer follow-up and direct microbiome and metabolomic assessment are needed to clarify potential vascular effects of FMT.

## 1. Introduction

Globally, alcohol-related liver disease (ALD) is among the most frequently encountered liver disorders and is characterized by a high risk of mortality, especially in patients with advanced disease [1]. The severity of the disease is closely linked to cumulative alcohol exposure, including intake volume, duration and drinking pattern, with marked individual variability [2]. While moderate consumption is defined as up to two drinks per day for men and one for women, sustained intake exceeding 40 g of ethanol daily represents a key risk factor for ALD development [3,4].

ALD encompasses a wide pathological spectrum, ranging from simple steatosis to progressive fibrosis, cirrhosis and hepatocellular carcinoma [5]. Steatosis, the earliest and most prevalent stage, results from alcohol-induced lipid accumulation in hepatocytes and is often asymptomatic [6]. However, approximately 25% of cases progress to steatohepatitis and subsequent fibrotic remodeling [4]. Over time, advancing fibrosis may lead to cirrhosis, characterized by increased intrahepatic vascular resistance, portal hypertension, and severe complications such as ascites, hepatic encephalopathy, variceal bleeding, and bacterial infections [7,8].

Fecal microbiota transplantation (FMT) is a therapeutic intervention that involves the transfer of intestinal microorganisms from healthy donors to recipients in order to re-establish microbial equilibrium, reduce dysbiosis-associated taxa and support the treatment of conditions related to gut microbiome disruption [9]. It has emerged as a therapeutic option explored across a broad spectrum of conditions, supported by a favorable safety profile and encouraging clinical outcomes [10]. Clinical applications extend beyond gastrointestinal pathology to include dysbiosis-related systemic disorders, such as cardiovascular and metabolic diseases, neuropsychiatric and autoimmune conditions, as well as dermatologic and renal manifestations [11].

In patients with alcohol-related liver cirrhosis, arterial stiffness plays a significant role, indirectly influencing quality of life and contributing to an increased risk of mortality and disease-related complications [12]. In this context, modulation of the gut microbiota may influence vascular stiffness. However, the possibility remains that these mechanisms may evolve independently, without exerting a measurable impact on the cardiovascular safety profile. This research direction remains insufficiently explored in human studies, despite the availability of experimental evidence from animal models suggesting a potential relationship between gut microbiome composition and the degree of arterial stiffness, as well as a possible effect of microbiota-modulating interventions on vascular dynamics [13,14,15].

Frequently, findings obtained under laboratory conditions do not fully translate to in vivo outcomes, highlighting the need to integrate complementary functional assessment methods, such as non-invasive oscillometric arterial stiffness assessment using arteriograph-based pulse wave analysis, into the comprehensive evaluation of the safety profile of microbiota-modulating therapies in patients with liver cirrhosis. At the same time, this approach also underscores the limitations of such interventions, particularly with regard to systemic or remote effects, which are rarely assessed directly.

The arteriograph additionally provides a quantitative and objective assessment of arterial stiffness in the setting of advanced liver disease [16,17]. Given the current lack of standardized and stable biomarkers capable of accurately reflecting vascular changes secondary to microbiota-targeted interventions, this analytical method becomes an indispensable tool for identifying vascular response patterns and for elucidating the systemic impact of FMT.

## 2. Materials and Methods

### 2.1. Study Population and Eligibility Criteria

This study included adult patients hospitalized at the Sibiu County Clinical Emergency Hospital, predominantly within the Department of Gastroenterology, with a confirmed diagnosis of alcohol-related liver cirrhosis. Patients were eligible irrespective of Child–Pugh classification. However, the requirement of objectively confirmed alcohol abstinence following fecal microbiota transplantation (FMT), verified through a close relative or caregiver, resulted in the exclusion of several candidates. Despite this selection process, no statistically significant differences in baseline characteristics were observed across Child–Pugh severity categories. Exclusion criteria comprised age under 18 years, cirrhosis of non-alcoholic etiology, uncertain or unvalidated diagnosis and the presence of concomitant oncological diseases or major traumatic conditions with potential impact on study outcomes. Patients who declined participation or failed to provide written informed consent were excluded, as were individuals presenting with coma or severe impairment of consciousness. Although not a predefined inclusion criterion, the final study cohort consisted exclusively of male patients, thereby reducing heterogeneity related to sex-specific biological variability. Following enrollment, all participants were managed according to a standardized protocol corresponding to their assigned study group.

Patients with liver cirrhosis selected for FMT (*n* = 6) underwent a comprehensive baseline evaluation, including clinical assessment and also vascular stiffness measurements. Subsequently, a full colonoscopy reaching the cecum was performed and fecal microbiota transplantation was administered at this anatomical level using material obtained from a healthy donor. FMT was delivered by colonoscopic instillation into the cecum because this represented the standardized administration route available at our institution during the study period. Capsule-based delivery was not implemented within the local clinical protocol at that time.

Post-procedural follow-up was conducted at approximately 1 month and 3 months, with minor variations depending on patient availability and institutional logistics. Each follow-up visit included a clinical examination, abdominal ultrasonography, and vascular stiffness measurement. Throughout the study period, alcohol intake and the use of any antibiotics, including rifaximin, were strictly prohibited.

The control cohort (*n* = 13) was managed according to standard therapeutic protocols, which included a normocaloric diet with balanced protein, lipid, and carbohydrate content, as well as beta-adrenergic blockers, hepatoprotective supportive agents and diuretic therapy. All participants underwent an initial comprehensive assessment (defined as baseline or time zero), comprising clinical evaluation, imaging studies, elastographic measurements and laboratory analyses. Follow-up evaluations were subsequently performed at approximately one month after baseline, with limited flexibility in the timing of reassessment, similar to that allowed for the intervention group. Throughout the study period, alcohol intake and the use of antibiotics were prohibited; however, rifaximin administration was allowed in the control group.

Patient allocation was not randomized and was based on clinical eligibility for FMT, logistical feasibility, and patient consent. No formal matching procedure was applied between the study groups. Differences in management between groups, including rifaximin use in the control group and differences in follow-up time points, were inherent to the clinical protocol and were not controlled through randomization.

### 2.2. Fecal Donor Selection and Preparation Procedure

Fecal donors were recruited either from among patients’ relatives or from unrelated volunteers. Both related and unrelated donors were considered eligible in the present pilot study due to practical donor availability constraints within the local clinical setting, provided that all screening criteria were fulfilled. Donors were selected based on young age, absence of comorbidities and the provision of written informed consent. Each candidate underwent an extensive clinical and laboratory screening process designed to minimize the risk of pathogen transmission. In addition, donors completed a standardized epidemiological questionnaire intended to identify and exclude individuals with potential infectious or transmissible risk factors relevant to patients with liver cirrhosis. On the day of transplantation, donors prepared the fecal suspension according to a standardized protocol provided at the time of donor approval. This protocol was adapted from the procedure routinely employed in the Gastroenterology Clinic of Sibiu for the management of Clostridioides difficile infection and was strictly adhered to in all cases. Thus, all FMT procedures were performed using the same preparation steps, stool quantity threshold, saline dilution, filtration method, bowel preparation protocol and colonoscopic instillation route. In brief, stool samples were collected on the morning of the procedure and processed within a maximum of six hours to preserve microbial viability. In accordance with the standardized protocol routinely used in our Gastroenterology Clinic, a minimum of 70 g of fecal material was homogenized with 250 mL of 0.9% sodium chloride solution for at least three minutes. This quantity was selected to ensure procedural consistency and adequate microbial biomass, although we acknowledge that lower stool quantities have also been used in other published protocols. The resulting suspension was subsequently filtered through a two- to three-layer gauze, and the filtrate constituted the final material used for transplantation. Colonoscopy was performed following standard bowel preparation with polyethylene glycol-based solutions and all procedures were conducted under analgosedation. To enhance retention of the transplanted material, patients were instructed to refrain from bowel evacuation for at least two hours after the procedure. No additional medications with potential influence on study outcomes were administered during the study period. No antibiotic pretreatment was administered prior to FMT. This decision was made in order to avoid additional pharmacological exposure and potential confounding effects in a clinically vulnerable cirrhotic population.

Donor screening included clinical evaluation, exclusion of epidemiological and clinical risk factors based on a standardized questionnaire, stool testing and blood-based infectious screening. Stool analyses included coproculture and Adler test. Blood tests included anti-HAV IgM, anti-HCV antibodies, HBsAg, anti-HBe, HBeAg, HIV testing and heterophile antibodies. Multiple donors were used in this pilot study depending on availability. Donor material was assigned pragmatically according to donor eligibility and procedural scheduling, rather than through a predefined allocation algorithm. No microbiome sequencing was performed to characterize donor profiles or donor–recipient matching.

For the assessment of vascular stiffness, an initial data collection was performed in patients diagnosed with alcohol-related liver cirrhosis at baseline (time point 0), prior to FMT. Measurements were conducted using an arteriograph under standardized and identical conditions for both study groups: the experimental group and the control group. The device employed was the Medexpert Arteriograph TL2 (Medexpert Ltd., Budapest, Hungary), used in conjunction with the dedicated Medexpert Arteriograph software (version 3.0.0.0), which enables the acquisition of a wide range of parameters with major relevance for cardiovascular function assessment.

Patients were clearly informed about the importance and purpose of the non-invasive investigation, as well as about the preparatory steps and procedural stages involved. Follow-up arteriographic assessments were performed approximately one month and three months after FMT, maintaining a consistent set of monitored parameters and identical examination conditions across all sessions. The control group was reassessed once, at one month following the baseline evaluation.

Arteriography requires minimal patient preparation, with most recommendations being similar to those applicable to standard blood pressure measurement. Specifically, patients were positioned in the supine position, in a comfortable posture, with the lower limbs uncrossed and with adequate support of the back and arm to ensure measurement accuracy. To ensure optimal examination conditions, the procedure was conducted in an environment protected from extreme temperature variations and excessive ambient noise.

Following a 10-min rest period, arm circumference was measured for each patient and the appropriate cuff size for pulse wave measurement was selected according to the recommendations of the dedicated arteriography software. The cuff was applied around the arm of the dominant upper limb, at an appropriate distance from the thorax, with the tubing oriented anteriorly. Proper cuff tightness was verified to ensure optimal measurement accuracy. Prior to initiating the actual measurement, the distance between the suprasternal notch and the pubic bone was measured using a measuring tape and the obtained value was entered into the software.

During data acquisition, patients were instructed to avoid any body movement and to refrain from speaking, in order to prevent motion artifacts that could compromise measurement accuracy. In the three hours preceding the examination, patients avoided the consumption of large meals, coffee intake and smoking. Alcohol consumption was prohibited for at least ten hours prior to the examination to prevent interference with the results.

The following hemodynamic parameters were calculated during each measurement:

- Sys—Brachial systolic blood pressure (mmHg);
- Dia—Brachial diastolic blood pressure (mmHg);
- HR—Heart rate (beats/min);
- MAP—Mean arterial pressure (mmHg);
- PP—Brachial pulse pressure (mmHg);
- Aix aortic—Central/aortic augmentation index (%).

These are calculated based on a very strong linear relationship (R > 0.9) between the brachial and central augmentation indices.

- Aix 75%—Augmentation index normalized to 75 bpm (%);
- ED—Left ventricular ejection duration (ms).

This represents the duration of mechanical systole, defined as the interval between aortic valve opening and closure.

- RT—Return/reflection time (ms).

This is the time required for the pulse wave to travel from the aortic root to the bifurcation and back.

- PWVao—Aortic pulse wave velocity (m/s).

This represents the propagation velocity of the pulse wave within the aorta. It is calculated by dividing the traveled distance (measured as the distance between the suprasternal notch and the pubic bone) by the measured transit time (RT/2).

- SD PWVao (m/s).

This is a parameter providing information on measurement quality. PWVao is calculated for each recorded pulse wave, and the standard deviation is subsequently displayed.

- PPao—Central pulse pressure (mmHg).

This is defined as the difference between aortic systolic and diastolic blood pressure values.

For each measurement, quality was assessed by monitoring the standard deviation (SD) value, ensuring that it remained within normal limits. In cases where qualitative errors were detected, patient preparation steps were repeated and the measurement was reperformed to ensure the acquisition of optimal and reliable results.

### 2.3. Statistical Analysis

All statistical analyses were performed using SPSS software, version 24.0. Continuous variables were expressed as median (IQR) or mean ± SD, depending on distribution. Group comparisons were performed using the Mann–Whitney U test or Student’s *t*-test, as appropriate. Categorical variables were compared using chi-square or Fisher’s exact test. Within-group longitudinal changes were assessed using repeated-measures analysis (Friedman test or repeated-measures ANOVA, as appropriate).

Given the exploratory nature of the study and the small sample size, results are presented descriptively and emphasis is placed on effect direction rather than statistical significance alone.

## 3. Results

### 3.1. Evolution of Arterial Stiffness Indices Before Transplantation and One Month After Transplantation: Comparison Between the Study Group and the Control Group

In the comparative analysis of vascular stiffness parameters between the study group and the control group at baseline (pre-transplantation), statistically significant differences were identified for two variables. Systolic duration was significantly shorter in the study group, with a median value of 0.24 (interquartile range [IQR] 0.14–0.30), compared with 0.45 (0.34–0.52) in the control group, the difference reaching statistical significance (*p* = 0.046). In addition, return time (RT) was significantly longer in the study group, with a median value of 126 ms (117–145), compared with 105 ms (96–125) in the control group, this difference also being statistically significant (*p* = 0.036). All other assessed indices showed no statistically significant differences between the two groups at baseline (Table 1).

At one month after FMT, no statistically significant differences in vascular stiffness indices were observed between the study and control groups. Aortic augmentation index showed a median value of 36.4% in the study group and 30.4% in the control group (*p* = 0.416), while augmentation index at 75 bpm was 30.5% versus 24.8% (*p* = 0.831). Return time was 132.5 ms in the study group and 122 ms in the control group (*p* = 0.210). Ejection duration, systolic duration, and aortic pulse wave velocity also showed no statistically significant between-group differences (Table 2).

### 3.2. Dynamics of Arterial Stiffness Indices in Patients Undergoing Fecal Microbiota Transplantation During the Study Period

The vascular stiffness indices analyzed in the group of patients undergoing FMT did not show statistically significant changes across the three evaluation time points:

• Pre-transplantation;
• At one month;
• At three months post-intervention (Table 3).

However, ED showed a numerical decrease at three months compared with baseline and one month, but this change did not reach statistical significance (*p* = 0.069). The remaining parameters, including Aix aortic, Aix 75%, systolic duration, PWVao and RT, did not exhibit statistically significant changes across the three evaluation time points.

## 4. Discussion

In the assessment of arterial stiffness, the arteriograph was used to record a broad range of parameters, some of which are directly correlated with vascular stiffness, while others exhibit more indirect associations [18,19]. When comparing the groups prior to FMT, several statistically significant differences were identified, particularly with respect to systolic duration and RT. The observed difference in systolic duration was located just below the conventional threshold of statistical significance (0.05), suggesting a relatively weak association, in contrast to the more pronounced difference observed for RT. Given this baseline discrepancy between groups regarding RT, this variable may represent a potential selection-related confounding factor. The absence of detectable changes in arterial stiffness should be interpreted cautiously and may reflect the limited duration of follow-up, the advanced disease stage and the exploratory nature of the study. Nevertheless, as no significant differences were identified for the remaining variables, intergroup comparisons remain justified (Table 1).

At the initial comparison between the control group and the experimental group at one-month post-intervention, PWVao, a key and direct marker of vascular stiffness, did not differ significantly from that observed in patients receiving standard treatment. A similar finding was noted for RT, another variable with direct relevance to arterial stiffness assessment. The remaining parameters, including the aortic augmentation index and ejection duration, variables indirectly associated with vascular stiffness, also showed no significant differences between the control and experimental groups, with *p*-values well above the threshold of statistical significance. In addition, an analysis restricted to the experimental group was performed to evaluate parameter variation throughout the study period. Among these variables, only ejection duration approached the threshold of statistical significance. However, this change lacks major clinical relevance when compared with parameters that directly reflect vascular stiffness.

At present, there are no clinical studies that clearly demonstrate the effects of FMT on vascular stiffness. Several years ago, Nathan Greenberg et al. demonstrated in an experimental animal model that age-associated aortic stiffness can be transferred and, at the same time, ameliorated through FMT in mice [20]. In another study, Rafael Cuadrat et al. reported a significant correlation between gut microbiota composition and vascular stiffness [13]. Increased gut microbial diversity was associated with reduced arterial stiffness [21]. However, these findings have not yet been validated in multicenter human studies and require further investigation.

In essence, the microbiota transfer applied in the present clinical study addresses, to a considerable extent, the issue of reduced microbial diversity, which is frequently compromised in patients with liver cirrhosis. Nevertheless, multiple additional factors may both limit the generalizability of the obtained results and substantially influence the potential impact of the microbiota on arterial stiffness. One possible explanation for the absence of significant changes in vascular stiffness is that, in patients with advanced alcohol-related cirrhosis, vascular alterations may be less responsive to short-term intervention. In this context, the present findings may indicate that any vascular effects of microbiota modulation, if present, are not readily detectable over a three-month follow-up period in this clinical setting.

One important explanation for the absence of significant vascular improvement may be related to the advanced stage of liver disease in the included cohort. In patients with long-standing alcohol-related cirrhosis, arterial stiffness is likely to reflect established structural vascular remodeling, endothelial dysfunction, and chronic hemodynamic alterations, which may not be readily reversible over a short follow-up period. Therefore, the present findings should not be interpreted as excluding a vascular effect of FMT in general, but rather as suggesting that such an effect may be difficult to detect in patients with advanced disease. Future studies should include patients at earlier stages of disease progression, in order to determine whether disease stage influences the vascular response to FMT.

An additional methodological aspect that may have influenced the findings is donor selection. In several cases, donors were recruited from among patients’ relatives, a strategy that was feasible within the local pilot-study setting but may have reduced the microbiological contrast between donor and recipient due to shared environmental exposures and partially overlapping microbial communities. This factor may have attenuated the biological effect of FMT. Future studies should preferentially use rigorously screened unrelated anonymous donors or standardized donor-bank material to maximize donor–recipient microbiota divergence.

Although microbial diversity initially depends on donor-related characteristics, over time it tends to adapt according to the recipient’s environment and specific physiological conditions. Consequently, it remains unclear whether periodic repetition of microbiota transfer would have been necessary to achieve a measurable beneficial effect or whether the monitoring and follow-up period was insufficient to capture meaningful vascular changes.

Recipient preparation may also have influenced the intervention effect. Unlike some FMT protocols that include antibiotic pretreatment to reduce the resident microbial load and facilitate donor microbiota engraftment, no such pretreatment was used in the present study. Although this approach was considered more appropriate for a clinically fragile cirrhotic cohort, it may have limited the magnitude of microbiota replacement and, consequently, the likelihood of detecting downstream vascular effects. In addition, the route of administration was limited to colonoscopic delivery according to local protocol. Alternative approaches, such as capsule-based FMT, may represent valuable options in future studies and could improve feasibility, standardization, and patient acceptability.

Patient profile represents a key determinant that may influence both the analytical process and the study outcomes, as coexisting comorbidities can limit the impact of the microbiota on the organs and physiological systems with which it interacts in a complex manner. From a pathophysiological perspective, the relationship between arterial stiffness and alcohol-related liver cirrhosis remains insufficiently elucidated. Current literature increasingly reports significant correlations, through various mechanisms, between hepatic steatosis and arterial stiffness, rather than between alcohol-related cirrhosis and vascular rigidity per se [22,23,24].

Moderate alcohol consumption was initially perceived as a cardiovascular protective factor; however, supporting evidence for this hypothesis has been insufficient and has not been confirmed by rigorous, well-designed studies [25]. Accordingly, recent comprehensive analyses synthesizing major studies published worldwide have failed to reach a clear and definitive conclusion regarding the impact of alcohol consumption on arterial stiffness [12,26].

**Figure 1:**
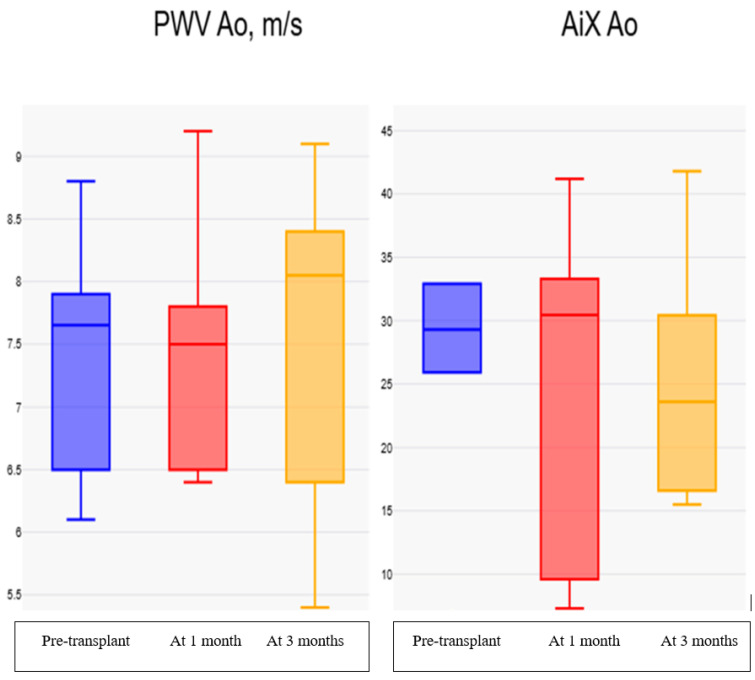
Evolution of the augmentation index and aortic pulse wave velocity in patients from the study cohort treated with FMT, at the three assessment time points: pre-transplant, at 1 month and at 3 months.

In the present pilot study, no statistically significant change in direct arterial stiffness parameters was observed during follow-up after FMT when compared with baseline values. Although small numerical variations were observed in selected parameters, these findings should be interpreted cautiously given the limited sample size, the absence of randomization, differences in management between groups, and the lack of direct verification of microbiota engraftment. Figure 1 illustrates the longitudinal evolution of augmentation index and aortic pulse wave velocity in the FMT group across the three assessment time points.

The study has several limitations. First, the relatively small number of enrolled patients restricts statistical power and limits the generalizability of the findings, although the present work was conceived as a pilot study intended to generate clinically relevant hypotheses for future investigations. Second, and most importantly, the study did not include direct assessment of microbiota modification through sequencing-based profiling, nor did it incorporate metabolomic markers capable of reflecting microbiota-related systemic effects. As a result, the biological impact of FMT cannot be confirmed directly, and the absence of vascular changes must be interpreted as a clinical observation rather than as evidence of absent microbiological activity. Future studies should integrate microbiome characterization together with metabolomic evaluation, including markers such as trimethylamine N-oxide (TMAO) and imidazole propionate, in order to verify engraftment, assess mechanistic pathways and better define the relationship between microbiota modulation and vascular function. Third, the use of related donors in part of the cohort and the absence of antibiotic pretreatment may also have attenuated donor–recipient microbiological contrast and reduced engraftment efficiency. Finally, the lack of multi-omics integration limited the exploration of complex biological interactions underlying the investigated pathology. Fourth, the lack of randomization and the presence of treatment-related differences between groups, including antibiotic exposure and follow-up structure, introduce potential confounding and limit the ability to draw causal inferences regarding the effect of FMT on vascular parameters.

## 5. Conclusions

In this prospective pilot study of patients with alcohol-related liver cirrhosis, FMT was not associated with statistically significant short-term changes in arterial stiffness parameters during the three-month follow-up period. Direct markers of vascular stiffness, including aortic pulse wave velocity and return time, remained broadly stable and did not show meaningful longitudinal variation after the intervention.

Although minor numerical fluctuations were observed in selected hemodynamic indices, these did not translate into consistent changes suggestive of a measurable vascular effect in this small cohort. Accordingly, the present findings do not support a detectable short-term impact of FMT on arterial stiffness under the conditions evaluated in this study. These results should be interpreted cautiously because of the pilot nature of the study, the limited sample size, the non-randomized design, differences in management between groups and the absence of direct microbiome or engraftment assessment. Arteriography nevertheless proved to be a feasible and objective method for vascular evaluation in this clinical setting.

Further studies involving larger cohorts, earlier disease stages, standardized donor selection, and integrated microbiome and metabolomic assessment are needed to determine whether microbiota modulation can influence vascular function in patients with alcohol-related liver disease.

## Figures and Tables

**Table 1:** Vascular stiffness indices in the study group compared with the control group, pre-transplantation.

Variable	Study (*n* = 6)	Control (*N* = 13)	*p*-Value
**Aortic augmentation index (Aix aortic), %**	27 (27–44.6)	30 (23–38)	0.521
**Augmentation index at 75 bpm (Aix 75%), %**	29 (27–31)	28 (21–34)	0.765
**Ejection duration, ms**	307.5 (290–322.5)	288 (274–303)	0.106
**Systolic duration**	0.24 (0.14–0.3)	0.45 (0.34–0.52)	**0.046**
**Aortic pulse wave velocity, m/s**	7.65 (7.2–8.25)	8.7 (7.6–10.1)	0.072
**Return time, ms**	126 (117–145)	105 (96–125)	**0.036**

**Table 2:** Comparison of vascular stiffness indices between the study group and the control group at 1 month after FMT.

Variable	Study	Control	*p*-Value
**Aortic augmentation index (Aix aortic), %**	36.4 (19.2–40.4)	30.4 (19.1–34)	0.416
**Augmentation index at 75 bpm (Aix 75%), %**	30.5 (14.5–32.9)	24.8 (18.2–30.9)	0.831
**Ejection duration, ms**	312.5 (305–320)	301 (297–310)	0.152
**Systolic duration**	0.4 (0.2–0.6)	0.4 (0.2–0.4)	0.966
**Aortic pulse wave velocity, m/s**	7.5 (6.7–7.8)	7.8 (7.1–9.4)	0.467
**Return time, ms**	132.5 (121.2–140)	122 (105–133)	0.210

**Table 3:** Vascular stiffness indices in the FMT group during the study period.

Variable	Study	1 Month	3 Months	*p*-Value
**Aortic augmentation index (Aix aortic), %**	27 (27–44)	36 (19–40)	24 (18–33)	0.846
**Augmentation index at 75 bpm (Aix 75%), %**	29 (27–31)	30 (14–32)	23 (16–31)	0.846
**Ejection duration, ms**	307 (290–322)	312 (305–320)	291 (246–300)	0.069
**Systolic duration**	0.24 (0.14–0.3)	0.4 (0.2–0.6)	0.39 (0.28–0.49)	0.607
**Aortic pulse wave velocity, m/s**	7.65 (7.2–8.25)	7.5 (6.7–7.8)	8.0 (7.9–8.6)	0.834
**Return time, ms**	126 (117–145)	132 (121–140)	122 (118–179)	0.513

## Data Availability

The data presented in this study are available from the corresponding authors upon reasonable request due to privacy, legal and ethical reasons.
